# XALIA: rationale and design of a non-interventional study of rivaroxaban compared with standard therapy for initial and long-term anticoagulation in deep vein thrombosis

**DOI:** 10.1186/1477-9560-12-16

**Published:** 2014-07-14

**Authors:** Walter Ageno, Lorenzo G Mantovani, Sylvia Haas, Reinhold Kreutz, Verena Haupt, Jonas Schneider, Alexander GG Turpie

**Affiliations:** 1 Department of Clinical and Experimental Medicine, University of Insubria, Via Guicciardini 9, Varese 21100, Italy; 2 Department of Clinical Medicine and Surgery, Università degli Studi di Napoli Federico II, Naples, Italy; 3 Vascular Centre, Normannenstr. 34a, Munich 81925, Germany; 4 Institute of Clinical Pharmacology and Toxicology, Charité-Universitätsmedizin, Berlin, Germany; 5 Bayer Vital GmbH, Leverkusen, K56, 51368, Germany; 6 Bayer Pharma AG, Muellerstr. 178 S101, Berlin 13353, Germany; 7 Department of Medicine, Hamilton Health Sciences, General Division, 237 Barton Street East, Hamilton, Ontario L8L 2X2, Canada

**Keywords:** Deep vein thrombosis, Outcomes, Real-world experience, Rivaroxaban

## Abstract

Venous thromboembolism (VTE), comprising deep vein thrombosis (DVT) and pulmonary embolism, poses a substantial clinical risk, and the incidence of these thrombotic-related diseases remains high. Anticoagulation aims to prevent thrombus extension and reduce the risk of recurrent events, particularly fatal pulmonary embolism. In EINSTEIN DVT, rivaroxaban was non-inferior to enoxaparin/vitamin K antagonists for the reduction of recurrent VTE, with a similar safety profile and a net clinical benefit. EINSTEIN EXT investigated patients receiving long-term treatment in whom there was no clear decision about continuing or stopping anticoagulation; rivaroxaban was superior to placebo in the reduction of recurrent VTE, showing an acceptable benefit–risk balance. Rivaroxaban has the potential to replace standard therapy, usually parenteral low molecular weight heparin overlapping with and followed by a vitamin K antagonist, for the treatment of acute symptomatic DVT and the secondary prevention of VTE. As the use of rivaroxaban for DVT treatment increases in clinical practice, a fundamental understanding of its clinical benefits in everyday patient care is essential. XALIA (XArelto for Long-term and Initial Anticoagulation in venous thromboembolism) is a multicentre, prospective, non-interventional, observational study investigating the effectiveness and safety of a single-drug approach with rivaroxaban compared with standard therapy in patients with DVT. The study cohort will include approximately 4800 patients (≥18 years old) with objectively confirmed acute DVT who will be treated for a period of ≥3 months. The primary outcomes will be the incidence of treatment-emergent adverse events (primarily major bleeding), symptomatic recurrent venous thromboembolic events and all-cause mortality. Secondary outcomes include: major cardiovascular events; patient-reported treatment satisfaction and adherence; healthcare resource utilization; reasons for drug switching or interruption of treatment; and adverse events. XALIA will follow an international cohort of patients in more than 20 European countries, and others including Israel and Canada. The first patient was enrolled in June 2012, with results expected in 2015. It is anticipated that XALIA will provide important information on the treatment of DVT in a heterogeneous, unselected patient population in a real-world setting and provide important supplementary information to that obtained from the EINSTEIN DVT phase III study.

## Introduction

Acute venous thromboembolism (VTE), which comprises deep vein thrombosis (DVT) and pulmonary embolism (PE), is the third most common cardiovascular disorder [1]. VTE occurs with an incidence of 1–2 cases per 1000 people in the general population and the risk increases with age [2,3]. Treatment of VTE involves an initial phase, in which the key imperative is to prevent thrombus extension, improve acute symptoms and reduce the risk of early PE [4]. The current standard treatment for acute VTE is overlapping anticoagulation with a parenteral agent (such as low molecular weight heparin [LMWH]) and a vitamin K antagonist (VKA) [5]. VKAs may be given over a period of months, years, or even indefinitely, to reduce the risk of recurrent events. Guidelines on the duration of anticoagulant therapy for VTE, including those published by the American College of Chest Physicians (ACCP), recommend at least 3 months’ treatment after a provoked or unprovoked event [5].

Anticoagulation with LMWH is rapid and effective, but the need for daily subcutaneous injections can be challenging. Heparin-induced thrombocytopenia may be a risk with LMWH use, with an incidence of ≤1% in medical and postoperative patients [6]. The limitations of VKAs in routine clinical practice are well documented: treatment with these agents requires regular monitoring to ensure patients remain within the therapeutic range (international normalized ratio 2.0–3.0), and a wide range of drug–drug and drug–food interactions may influence therapeutic levels of anticoagulation. Initial adjustment of VKA dosing in the first weeks of therapy is particularly challenging and in some cases is not easy to achieve [7]. Furthermore, the use of a dual-drug approach with LMWH and VKAs can be both complex and burdensome for patients and healthcare providers.

Direct oral Factor Xa inhibitors, such as apixaban, edoxaban and rivaroxaban, and the direct oral thrombin inhibitor dabigatran, have recently been investigated in phase III clinical trials for the treatment and secondary prevention of DVT and PE [8-15]. Rivaroxaban and apixaban have both been studied as a single-drug approach, whereas edoxaban and dabigatran employed an initial course of parenteral heparin therapy before commencing therapy with the anticoagulant being investigated. Data show that these agents are effective and well tolerated; however, it is not known whether these findings are directly applicable to patients in a real-world setting. As the use of the direct oral anticoagulants in clinical practice increases, a fundamental understanding of their efficacy and safety in patients with VTE treated in routine care is essential. XALIA (XArelto for Long-term and Initial Anticoagulation in venous thromboembolism) will investigate the effectiveness of a single-drug approach with rivaroxaban compared with standard dual-drug therapy with LMWH/VKAs in the real-world treatment of patients with DVT.

### Rivaroxaban

Rivaroxaban is a direct oral Factor Xa inhibitor with a high oral bioavailability (≥80%) [16] and predictable pharmacokinetics and pharmacodynamics [17,18]; these characteristics provide the basis for use of this agent without the need for routine coagulation monitoring. The onset of action of rivaroxaban, i.e. its anti-Factor Xa activity, has been shown to be as fast as that of the LMWH enoxaparin [19]. Peak plasma concentrations of rivaroxaban are achieved within 2–4 hours and it has a mean half-life of approximately 9 hours (5–9 hours in young individuals and 11–13 hours in the elderly) [17,18]. Rivaroxaban is associated with few drug–drug [20-22] or drug–food interactions [23]. However, rivaroxaban doses of 15 mg and 20 mg should be taken with food to maintain high oral bioavailability [23].

XALIA is being conducted in response to a request made by the European Medicines Agency (EMA) to evaluate rivaroxaban for the treatment of acute DVT after the completion of the large phase III EINSTEIN DVT study. The EINSTEIN phase III clinical programme investigated the use of rivaroxaban as a single-drug approach in contrast to the current dual-drug paradigm of enoxaparin/VKAs [8,9]. EINSTEIN DVT was a randomized, open-label, event-driven, non-inferiority trial that investigated the efficacy and safety of oral rivaroxaban (15 mg twice daily for 21 days followed by 20 mg once daily) compared with subcutaneous enoxaparin (1 mg/kg body weight twice daily for at least 5 days) overlapping with and followed by a VKA (warfarin or acenocoumarol, started within 48 hours after randomization), in patients with acute, symptomatic, objectively confirmed proximal DVT without PE. Treatment duration was at the discretion of the attending physician and was planned for 3, 6 or 12 months [8]. In EINSTEIN DVT, rivaroxaban was shown to be non-inferior to enoxaparin/VKA for the reduction of recurrent VTE (Table 1) [8]. Rates of first major or non-major clinically relevant bleeding (the primary safety outcome) were similar in patients receiving rivaroxaban compared with patients receiving enoxaparin/VKA. In addition, rivaroxaban was associated with a significantly improved net clinical benefit (defined as the composite of symptomatic, recurrent VTE and major bleeding), compared with enoxaparin/VKA (Table 1) [8]. The consideration of patient-reported outcomes is becoming increasingly important when evaluating the overall effectiveness of direct oral anticoagulants. It is worth noting that an EINSTEIN DVT subanalysis showed that rivaroxaban was associated with improved treatment satisfaction compared with enoxaparin/VKA therapy in patients with DVT [24]. Among the factors noted were a reduction in patient-reported anticoagulation burden and an increase in convenience [24]. It would, therefore, be desirable to confirm the influence of rivaroxaban treatment on patient lives in routine care.

**Table 1:** Efficacy and safety outcomes of the phase III EINSTEIN DVT study

	**Rivaroxaban**	**Enoxaparin/VKA**	**HR (95% CI)**	**P value**
**Efficacy outcomes (ITT population)**	(n = 1731)	(n = 1718)		
Recurrent VTE* (%)	2.1	3.0	0.68 (0.44–1.04)	<0.001
**Safety outcomes (safety population)**	(n = 1718)	(n = 1711)		
First major or non-major clinically relevant bleeding^†^ (%)	8.1	8.1	0.97 (0.76–1.22)	0.77
Major bleeding (%)	0.8	1.2	0.65 (0.33–1.30)	0.21
Non-major clinically relevant bleeding (%)	7.3	7.0		
Total mortality (%)	2.2	2.9	0.67 (0.44–1.02)	0.06
Any treatment-emergent adverse event (%)	62.7	63.1		
**Net clinical benefit**	2.9	4.2	0.67 (0.47–0.95)	0.03
**(composite of symptomatic, recurrent VTE plus major bleeding, %)**				

*Primary efficacy outcome; ^†^principal safety outcome.

CI, confidence interval; DVT, deep vein thrombosis; HR, hazard ratio; ITT, intention to treat; PE, pulmonary embolism; VKA, vitamin K antagonist; VTE, venous thromboembolism.

The EINSTEIN PE study investigated rivaroxaban in patients with acute, symptomatic PE, and also showed that it was non-inferior to enoxaparin/VKA, with a 51% reduction in the incidence of major bleeding [9]. The EINSTEIN EXT study investigated rivaroxaban in patients receiving long-term treatment in whom there was no clear decision about continuing or stopping anticoagulation; rivaroxaban was superior to placebo in the reduction of recurrent VTE, and showed an acceptable benefit–risk balance [8].

Rivaroxaban is the first direct oral anticoagulant to be approved for the treatment of DVT and PE and secondary prevention of VTE in the European Union, US and Canada. XALIA is a multicentre, prospective, non-interventional, observational study taking place across more than 20 European countries, and other countries including Israel and Canada. XALIA will assess whether the results obtained with rivaroxaban in real-world clinical practice in patients with objectively confirmed, acute DVT and an indication to receive anticoagulation for at least 3 months are consistent with those seen in the phase III EINSTEIN DVT study.

The main objective of the study is to evaluate the safety of rivaroxaban in routine clinical practice for the treatment of acute symptomatic DVT and prevention of recurrent VTE by examining the incidence of treatment-emergent adverse events: major bleeding, symptomatic recurrent venous thromboembolic events and all-cause mortality.

## XALIA study design

The study cohort consists of patients with acute DVT treated with rivaroxaban or current standard therapy (initial treatment with unfractionated heparin, LMWH or fondaparinux, which may overlap with and be followed by an oral VKA) for a period of ≥3 months. The study duration will be 12 months from the final date of patient enrolment, but the treatment duration for each patient will be determined solely by the attending physician and does not depend on the initial intended treatment duration or the study duration. The first patient was enrolled in June 2012 and the end of study is expected in 2015.

### Administrative organization

Non-interventional post-authorization safety studies are recommended for review by Ethics Committees, and the study/sponsor sought approval from an independent Ethics Committee or Institutional Review Board in all countries where such procedures were in place.

An external expert scientific Steering Committee will give support regarding study objectives, study conduct and analysis. An independent Adjudication Committee blinded to the treatment group will adjudicate events. Members of the Steering Committee may also serve on the Adjudication Committee; however, no sponsor representatives will serve on the Adjudication Committee. The study is registered at clinicaltrials.gov (NCT01619007).

### Centre and patient selection

Recruitment of patients will be conducted by investigators at selected study centres in Europe and other regions in which rivaroxaban is approved. Enrolment is planned for approximately 4800 patients with approximately equal numbers in each arm. Patients can be enrolled immediately after a decision about the choice of anticoagulant therapy has been made, but no randomization will be undertaken. Inclusion and exclusion criteria are summarized in Table 2.

**Table 2:** Inclusion and exclusion criteria for XALIA

**Inclusion**	**Exclusion**
● Female or male patients who are ≥18 years of age	● Patients with acute symptomatic PE*
● Diagnosis of acute DVT, objectively confirmed	● Other exclusion criteria are dependent on local product information
● Indication for anticoagulation therapy for at least 12 weeks	
● Patients willing to participate in the study and available for follow-up	

*A parallel symptomatic PE is not an exclusion criterion; however, XALIA will not enrol patients with only PE.

DVT, deep vein thrombosis; PE, pulmonary embolism.

Patients who are ≥18 years old, with a diagnosis of objectively confirmed, acute DVT and an indication to receive anticoagulation for ≥3 months will be included. If anticoagulation is administered for a shorter period than planned, patients will remain in the study.

### Study outcomes

Primary study outcomes are the incidence of treatment-emergent adverse events, namely major bleeding, symptomatic recurrent venous thromboembolic events and all-cause mortality. Major bleeding is defined as overt bleeding associated with a fall in haemoglobin of ≥2 g/dl; or a transfusion of ≥2 units of packed red blood cells or whole blood; or bleeding at a critical site (intracranial, intraspinal, intraocular, pericardial, intra-articular, intramuscular with compartment syndrome, retroperitoneal); or death. Secondary outcomes include: major cardiovascular events; patient-reported treatment satisfaction and adherence; healthcare resource utilization; reasons for drug switching or interruption of treatment; and adverse events, including serious adverse events. Data on patients with renal impairment treated for acute DVT within the terms of the local product information will also be collected and analysed. The Adjudication Committee, blinded to the treatment group, will adjudicate bleeding events; recurrent VTE and other thromboembolic events; major cardiovascular events; and cause of death. Local objective diagnosis of symptomatic recurrent venous thromboembolic events is a prespecified criterion for event adjudication.

### Drug administration

The type, dose and duration of drug therapy are at the sole discretion of the attending physician. Enrolment will only be considered after the treatment decision has been made. Patients who commence treatment with rivaroxaban, or those who initially receive treatment with heparin/fondaparinux within 48 hours of enrolment, can be included in the rivaroxaban cohort. Patients in the standard-of-care cohort are those receiving other recommended pharmacological treatments for VTE according to international guidelines, e.g. initial unfractionated heparin, LMWH or fondaparinux, which may overlap with and be followed by an oral VKA. However, in routine clinical practice many patients for whom rivaroxaban is planned may initially receive parenteral anticoagulants for a period longer than 48 hours before they are switched to rivaroxaban. These patients will be excluded from the main analysis, but the allocation of these patients will be subject to sensitivity tests as follows: 1) they will be analysed separately and 2) they will be allocated to standard of care or rivaroxaban dependent on the distribution of days (after the first 48 hours) in which they actually switched to rivaroxaban; the cut-off points that will be used for these sensitivity analyses will be defined by the quartiles of the distribution of days (after the 48 hours) in which the switch occurs.

Concomitant medications taken prior to or during the study will be documented and include non-steroidal anti-inflammatory drugs, acetylsalicylic acid, clopidogrel, steroids, medications known to interact with the DVT treatment, and medications known to increase or reduce the incidence of DVT (e.g. hormone therapy). Other drugs will be recorded if considered medically relevant by the investigator. Any changes to VTE treatment, including switching or cessation of treatment, will be documented.

### Data acquisition

All patient data will be collected during the initial visit and routine follow-up visits (occurring around month 1, quarterly and at final assessment) or by mail, telephone or email (Table 3). Demographic data and clinical characteristics will be collected from medical records if available, or by patient interviews. Patient-reported treatment satisfaction will be assessed using a specific treatment satisfaction questionnaire: the validated Anti-Clot Treatment Scale (ACTS), as used in the EINSTEIN DVT and EINSTEIN PE studies [24,25].

**Table 3:** Overview of data collection procedures

**Scheduled procedure**	**Initial visit (baseline)**	**After 1 month**	**Quarterly**	**Final assessment**
Visit date	X	X	X	X
Demographic data	X			
Medical/surgical history	X			
Concomitant diseases	X		X*	X*
Concomitant medication/treatment	X		X*	X*
Physical examination	X			
Laboratory results (if available)	X	X	X	X
Diagnosis of current DVT	X			
Previous VTE	X			
Current VTE treatment regimen	X		X	X
Initial phase treatment		X		
Dose reduction (twice daily to once daily)		X		
Assessment of therapy				X
Patient satisfaction and adherence with treatment (optional, only in a sub-sample of patients)		X*	X*	X*
Adverse events^†^		X	X	X

*Only documented if new information is available from regular practice. No additional diagnostics are required for the study; ^†^serious adverse events must be reported to the sponsor within 24 hours.

DVT, deep vein thrombosis; VTE, venous thromboembolism.

### Statistical analysis

The primary statistical analysis includes a descriptive analysis of the primary and secondary outcomes. The primary outcome variable is the incidence of treatment-emergent adverse events, namely major bleeding and symptomatic recurrent VTE. An adverse event is considered as treatment emergent if it starts on or after the day of the first dose of study medication and ≤2 days after the last dose. All patients who took at least one dose of medication will be included in the safety population. For primary and secondary variables, incidence proportions and incidence rates will be estimated, together with 95% confidence intervals. The accepted range for the 95% confidence intervals is estimated to be between the allocation ratios of 1:3 and 3:1; therefore, at least 25% of patients at each study centre should receive either standard of care or rivaroxaban therapy. To compare the treatment arms, the risk difference and corresponding 95% confidence interval will be calculated, and time-to-event analyses will be conducted.

The potential for allocation bias in open-label, non-randomized studies will be minimized by also presenting a propensity score analysis to control the effect of unbalanced covariates [26,27]. To minimize selection bias, physicians will be asked to document patients consecutively. To decrease the possibility of reporting bias, source data will be verified at approximately 10% of study centres in countries where this is legally possible. A sample size of 4800 patients assumes that 17% of patients will be non-evaluable because of non-overlapping propensity scores or because of withdrawn informed consent forms.

## Discussion

The demand for real-world data in addition to clinical trial data is increasing. Non-interventional studies are crucial for obtaining a different perspective on the safety and effectiveness of drugs when used in routine clinical practice. Unlike registry data, which is typically analysed retrospectively, studies such as XALIA are designed to assess prospectively defined outcomes of interest thus eliminating potential bias. It is anticipated that XALIA will provide important information on the treatment of DVT in a heterogeneous patient population in a real-world setting. Strict inclusion and exclusion criteria in randomized clinical trials, such as EINSTEIN DVT, may restrict the population in which therapies can be studied, and it is important to show that the findings from clinical trials are applicable to patients treated in routine clinical practice.

One challenge with open-label observational studies is the potential for increased reporting of adverse events with the new drug, otherwise known as the Weber effect [28]. This may be a result of increased patient exposure and higher interest in the drug; however, when physicians become familiar with the drug and its adverse event profile, the rate of reported adverse events usually declines [28]. Therefore, it may be anticipated that this effect will also occur with rivaroxaban, at least during the initial period after its approval for the treatment of VTE.

Findings from XALIA may enable healthcare professionals to gain new insights into the management of patients with DVT, particularly regarding the risks of bleeding and recurrent VTE. Patients are at an immediate and continued risk of recurrence after the first venous thromboembolic event [29], and this risk can be substantial after cessation of anticoagulation [30,31]. Real-world data on recurrence rates will, therefore, be meaningful, particularly in patients who may discontinue anticoagulation. In some cases, the optimal duration of anticoagulation therapy for DVT is unclear; this may be partly owing to challenges in maintaining an appropriate benefit–risk balance because of an increased risk of bleeding or a patient’s intrinsic risk factors [5]. It is hoped that by evaluating the incidence and type of thromboembolic events and bleeding events in XALIA, an accurate representation of the benefit–risk profile of rivaroxaban in routine care will be provided.

The clinical impact of anticoagulant-related bleeding complications is often severe. The case–fatality rate for major bleeding in patients with VTE has been reported to be as high as 11% within the first 3 months [32]. Furthermore, data from the RIETE registry showed that 56% of deaths in patients with VTE who received anticoagulation for 3 months were as a result of bleeding [33]. As mentioned previously, clinical trials are selective in nature, and may underestimate the frequency of bleeding seen in clinical practice. Results from a large community-based trial in the US showed an annual incidence of major bleeding as high as 12–13% in patients with VTE [34]. XALIA will provide valuable information on the incidence of major bleeding events that may occur with rivaroxaban or standard therapy in patients with DVT.

## Conclusions

XALIA is a large, non-interventional study investigating the safety of rivaroxaban compared with standard of care for the treatment of acute DVT in routine clinical practice. Real-world data in a large cohort across multiple countries will be a valuable addition to the results of the EINSTEIN programme. The study was started in June 2012, will complete recruitment in early 2014, and results are expected in 2015.

## Competing interests

WA has participated in advisory boards for Bayer HealthCare, Bristol-Myers Squibb (BMS), Pfizer and Daiichi Sankyo, and has received travel or research support from Bayer HealthCare, GlaxoSmithKline, Alexion Pharmaceuticals and Boehringer Ingelheim. LGM has been a consultant for Bayer HealthCare Pharmaceuticals and has received grants from Boehringer Ingelheim, Pfizer, BMS and Daiichi Sankyo. SH has been a consultant for Bayer HealthCare Pharmaceuticals, BMS, Boehringer Ingelheim, Daiichi Sankyo, Pfizer and Sanofi. RK has been a consultant for Bayer HealthCare, Berlin-Chemie, BMS and Daiichi Sankyo. AGGT has been a consultant for Bayer HealthCare, Janssen Pharmaceutical Research & Development, LLC, Astellas, Portola and Takeda. VH and JS are both employees of Bayer HealthCare.

## Authors’ contributions

All authors contributed to the study concept, design and implementation, and to the content and development of this manuscript. All authors read and approved the final manuscript.
